# Hemophagocytic Lymphohistiocytosis: An 18-Year-Long Retrospective Single-Center Study

**DOI:** 10.7759/cureus.88831

**Published:** 2025-07-26

**Authors:** Madalina Bota, Marius Colceriu, Cristina Blag

**Affiliations:** 1 Department of Mother and Child, Iuliu Hatieganu University of Medicine and Pharmacy, Cluj-Napoca, ROU; 2 Department of Pediatric Oncology and Hematology, Emergency Hospital for Children, Cluj-Napoca, ROU

**Keywords:** anakinra, cancer, hemophagocytic lymphohistiocytosis, infection, rituximab

## Abstract

Introduction: Hemophagocytic lymphohistiocytosis (HLH) is a disease characterized by systemic hyperinflammation, a so-called cytokine storm that leads to life-threatening complications. Up to this date, there is only one protocol available for patients with this pathology, and there are no second-line therapy protocols in cases of relapsed or refractory diseases.

Materials and methods: This is a retrospective observational study of patients treated in the Department of Pediatric Hematology and Oncology, Pediatric Clinic No. 2, Emergency Hospital for Children, in Cluj-Napoca, Romania, between 2006 and 2024.

Results: The population was represented by 23 patients, with two genetically confirmed familial HLH. The majority of the patients in the secondary HLH were due to Epstein-Barr virus (EBV) infection. Clinical symptoms were mainly represented by fever. Biologically, all patients had confirmed hemophagocytosis on bone marrow smear, while the majority presented with hyperferritinemia and cytopenias. The median time range from onset to diagnosis was 14 days. All patients were treated with the HLH-2004 protocol, and 17 received only induction, while others received continuation therapy plus associated therapies, such as antithymocyte globulin (ATG), rituximab, and anakinra. None of the patients underwent hematopoietic stem cell transplantation. We had a survival rate of 69.56%; five patients died, four with progressive disease and one due to complications.

Conclusion: HLH is considered a life-threatening disease; urgent diagnosis of both the disease and the underlying cause and treatment are essential for ensuring the best prognosis. Even so, there still are refractory diseases that need to be further studied in order to implement new therapeutic strategies.

## Introduction

Hemophagocytic lymphohistiocytosis (HLH) is a disease characterized by systemic hyperinflammation, a so-called cytokine storm that leads to life-threatening complications [1,2]. It is classified into two major forms: familial HLH (with UNC13D, STXBP2, PRF1, STX11 mutations) [1-4] and secondary HLH. Secondary HLH has a wide variety of causes, including infections (Epstein-Barr virus (EBV), cytomegalovirus (CMV), human immunodeficiency virus (HIV), malaria [5-7]), rheumatological diseases (juvenile idiopathic arthritis (JIA) [3]), malignancy [8], and even immunodeficiency syndromes [3].

Diagnostic criteria according to the HLH-2004 [1,2,4,8,9] include molecular diagnosis, functional cellular findings consistent with familial HLH, or five out of the eight clinical and biological criteria, such as fever, cytopenias, hypertriglyceridemia, hyperferritinemia, hypofibrinogenemia, hemophagocytosis, low or absent natural killer (NK)-cell activity, or high sCD25.

Up to this date, there is only one protocol available for patients with this pathology, and there are no well-established second-line therapy protocols in cases of relapsed or refractory diseases. Hematopoietic stem cell transplantation (HSCT) is recommended in primary cases [4] and severe secondary patients, but remission has to be priorly achieved. Several options of treatment [4] are under study, including anti-interferon gamma (anti-IFN gamma), such as emapalumab [10,11]; anti-Janus kinase (anti-JAK) 1/2 drugs, such as ruxolitinib [12]; or even anti-interleukin-1 (anti-IL-1) agents such as anakinra [1,13]. Studies show their efficacy in isolated situations, but the drugs are not available or approved in every country.

This is a retrospective descriptive study that aims to outline the clinical characteristics, treatment strategies, and outcomes of pediatric patients with familial and secondary HLH treated at our center over an 18-year period.

## Materials and methods

Study design

This is a retrospective descriptive study of patients treated in the Department of Pediatric Hematology and Oncology, Pediatric Clinic No. 2, Emergency Hospital for Children, in Cluj-Napoca, Romania, between 2006 and 2024.

Inclusion and exclusion criteria

Patients who fulfilled the HLH-2004 criteria were considered eligible for the study. In contrast, patients who did not fulfill the criteria were excluded from the study.

The HLH-2004 criteria establish the diagnosis if one of either 1 or 2 is fulfilled: (1) a molecular diagnosis consistent with HLH and (2) five out of the eight criteria are fulfilled: for clinical criteria, fever and splenomegaly; for laboratory criteria, cytopenias affecting ≥2 lineages in the peripheral blood (hemoglobin <90 g/l, platelets <100×10^9^/l, neutrophils <1×10^9^/l), hypertriglyceridemia (≥265 mg/dl) and/or hypofibrinogenemia (≤1.5 g/L), low or absent NK-cell activity, ferritin ≥500 mcg/L, and soluble CD25 ≥2400 U/ml; and for histopathological criteria, hemophagocytosis in the bone marrow, spleen, or lymph nodes.

The genetic testing was performed by whole-exome sequencing for all tested patients. Not all patients were tested for genetic alterations due to the lack of availability at the specific timepoint.

Patients were classified into familial and secondary HLH. Those who did not benefit from genetic testing but fulfilled five out of the eight criteria were classified into secondary HLH.

Data collection and statistical analysis

The data were collected from the patients' charts. Data collection from the charts was consistent with the criteria for HLH; all patients fulfilled the criteria. However, due to the long time length of the study, there were inconsistencies in data for all patients. Statistical analysis was made in Microsoft Office Excel (Microsoft Corp., Redmond, WA, USA); all results are descriptive. We did not perform comparison studies due to the small number of patients and high variability of clinical criteria, biological testing, and treatment possibilities. No inferential analyses were made due to the small population analyzed.

## Results

Twenty-three patients were considered eligible for inclusion in the study according to the HLH-2004 criteria. The majority (60.86%) of patients were male patients, and the median age was 11 years (age range: two months-23 years).

According to literature, patients were classified into familial and secondary HLH. We had two confirmed cases with genetic mutations characteristic for HLH, one with UNC13D and one with STXBP2, who also had EBV infection. There was one case that was suspected of Griscelli syndrome but could not be confirmed genetically, due to the lack of genetic testing at that timepoint. 

According to the criteria, 21 out of 23 patients (91.3%) were classified with secondary HLH, but not all of them had the benefit of genetic testing to exclude possible associations of genetic alterations. Patients with secondary HLH had variable causes, but most of the patients (11/23, 47.82%) were confirmed with EBV infection by serology. We had two cases (8.69%) associated with JIA, one case (4.34%) due to HIV confirmed by serology and Western blot and one case (4.34%) with confirmed malaria, confirmed by rapid test and thick blood smear, who also had EBV infection. There were three cases (13%) associated with malignancy. One patient had an onset of HLH and was later confirmed with non-Hodgkin lymphoma (NHL). The other two cases, one with acute lymphoblastic leukemia (ALL) and one with Wilms tumor, developed HLH during treatment. There were five cases (21.73%) with no confirmed cause (see Table 1).

**Table 1:** Etiology of HLH in our cohort F: female; M: male; HLH: hemophagocytic lymphohistiocytosis; EBV: Epstein-Barr virus; JIA: juvenile idiopathic arthritis; ALL B: acute lymphoblastic leukemia with B-cell precursors; NHL-DLBCL: non-Hodgkin lymphoma-diffuse large B-cell lymphoma; HIV: human immunodeficiency virus

No.	Age (years)/sex	Genetic testing (whole-exome sequencing)	Type of HLH
1	13, F	Yes, negative	Secondary, no etiology confirmed
2	15, M	No	Secondary, no etiology confirmed
3	17, M	No	Secondary, EBV infection
4	15, M	No	Secondary, no etiology confirmed
5	11, M	No	Secondary, EBV infection
6	4, M	Yes, negative	Secondary, EBV infection
7	17, M	No	Secondary, EBV infection
8	5, F	No	Secondary, JIA
9	14, F	No	Secondary, EBV infection
10	0.5, F	Yes, negative	Secondary, no etiology confirmed
11	8, M	Yes, negative	Secondary, JIA
12	16, M	No	Secondary, EBV infection
13	6, M	Yes, negative	Secondary, no etiology confirmed
14	5, F	No	Secondary, EBV infection
15	3, M	No	Secondary, EBV infection
16	0.2, M	Yes, positive	Familial (UNC13D mutation)
17	10, F	Yes, positive	Familial (STXBP2 mutation + EBV infection)
18	17, M	No	Secondary, EBV + *Plasmodium falciparum* infection
19	4, M	No	Secondary, malignancy, Wilms tumor
20	23, F	No	Secondary, malignancy, NHL-DLBCL
21	6, F	No	Secondary, malignancy, ALL B
22	14, F	No	Secondary, EBV infection
23	14, M	No	Secondary, HIV infection

Regarding clinical criteria, 20 patients (86.95%) presented with fever, and 17 patients (73.91%) presented with splenomegaly.

Biological criteria showed that all patients had confirmed hemophagocytosis on bone marrow smear and all presented with at least one-lineage cytopenia (as shown in Table 2). Twenty-one out of 23 patients (91.3%) presented with low hemoglobin level for their age, 78.26% had low platelet count, and 56.52% presented with low white blood cell (WBC) count. Leucocytosis was present in 6/23 patients (26.08%).

**Table 2:** Biological parameters *Bolded parameters are abnormal for age and sex. F: female; M: male; N/A: not applicable; WBC: white blood cell; PLT: platelet

No.	Age (years)	Sex	WBC (/µl)	Hemoglobin (g/dl)	PLT (/µl)	Ferritin (ng/ml)	Triglycerides (mg/dl)	Fibrinogen (mg/dl)
1	13	F	1440	8.3	68000	1255	445	195
2	15	M	2400	9	59000	4000	446	45
3	17	M	5900	10.3	69000	20000	175	214
4	15	M	20800	10.5	43000	2000	127	153
5	11	M	2000	8.9	61000	3374	391	207
6	4	M	45400	8.6	315000	8115	525	244
7	17	M	25300	12	207000	2027	497	363
8	5	F	21200	9.8	37000	2881	1182	85
9	14	F	3350	6.9	22000	2500	247	69
10	0.5	F	3620	3	62000	4393	237	84
11	8	M	5000	11.6	120000	7879	305	218
12	16	M	12640	11.1	223000	13389	431	219
13	6	M	39300	14.6	137000	9104	158	150
14	5	F	42000	10.7	59000	4638	713	49
15	3	M	1800	9	47000	3220	356	58
16	0.2	M	9200	8.9	63000	873	423	198
17	10	F	3170	10.5	65000	449	248	622
18	17	M	4640	9.7	27000	3369	256	413
19	4	M	12600	8.5	255000	20000	N/A	N/A
20	23	F	2300	10	104000	N/A	228	234
21	6	F	2200	12.5	102000	812	124	266
22	14	F	900	4.3	15000	6	41	135
23	14	M	1240	10.2	292000	29	123	280

Twenty-two patients were tested at diagnosis for hyperferritinemia, hypertriglyceridemia, and hypofibrinogenemia. Out of 22 patients, 20 (90.9%) had high levels of ferritin, 11 (50%) high levels of triglycerides, and only six (27.27%) low levels of fibrinogen (as shown in Table 2). IL-2 receptor was only tested in five patients, but all of them had levels above 2400 U/L. No patient benefited from NK-cell activity testing.

The time range between the onset of symptoms and the confirmed diagnosis was a median of 14 days (5-930 days).

Seven patients presented at diagnosis with organ failure, mainly hepatic and renal failure, but there were also cases with pancreatic and pulmonary involvement. We had two patients with septical status and one patient with associated autoimmune hemolytic anemia (AIHA).

All patients were treated according to the HLH-2004 protocol (see Table 3); 17 patients received only induction. Six patients needed continuation therapy, three with secondary HLH due to EBV, one who had no confirmed cause, and two with confirmed genetic mutations for familial HLH. We had a total of four cases with associated therapies: one with antithymocyte globulin (ATG), one with rituximab, one who received both ATG and rituximab, and one who received anakinra (see Table 4). Associated therapies were considered in cases refractory to induction and continuation therapy, with the aim of inducing remission in order to consolidate with HSCT. Rituximab was used in both situations with confirmed EBV infections and was administered once a week, for four weeks, at a dose of 375 mg/m^2^. ATG was used in both cases with familial HLH at a dose of 5 mg/kg. Anakinra was used at a dose of 10 mg/kg/day, in one patient with secondary HLH, but no confirmed etiology, with severe refractory disease, with initial partial remission, but with a severe and fast relapse leading to the patient's death. None of the patients was transplanted.

**Table 3:** Treatment protocols in induction and continuation therapies *Dose was adjusted according to the level of cyclosporinemia that was aimed at 200 mcg/L.

Treatment protocol	Medication	Dosage	Administration
Induction therapy (weeks 1-8)	Dexamethasone	10 mg/m^2^/day	Daily for 2 weeks
		5 mg/m^2^/day	Daily for 2 weeks
		2.5 mg/m^2^/day	Daily for 2 weeks
		1.25 mg/m^2^/day	Daily for 2 weeks
	Etoposide	150 mg/m^2^/adm	2 administration/week, for 2 weeks
			1 administration/week, for 6 weeks
	Cyclosporine	6 mg/kg/day starting dose*	Daily for 8 weeks
Continuation protocol (weeks 9-24)	Dexamethasone	10 mg/m^2^/day	3 days/week
	Etoposide	150 mg/m^2^/adm	1 administration/week
	Cyclosporine	6 mg/kg/day starting dose*	Daily

**Table 4:** Therapies according to types and causes of HLH HLH: hemophagocytic lymphohistiocytosis; ATG: antithymocyte globulin

Therapy	No. of patients	Type of HLH
HLH-2004 protocol	23/23	
Only HLH-2004	19/23	
Only induction protocol	17/19	Secondary
Induction + continuation protocol	2/19	Secondary
HLH-2004 + other therapy	4/23	
Rituximab	1/4	Secondary
ATG	1/4	Primary
Rituximab + ATG	1/4	Primary
Anakinra	1/4	Secondary

Nine patients needed dose reductions or omissions of drug administration due to their clinical and biological status. There were several complications during the treatment, as shown in Table 5.

**Table 5:** Complications during the treatment HLH: hemophagocytic lymphohistiocytosis; EBV: Epstein-Barr virus

Patient no./age	Etiology	Complication
Case 1, 13 years	No confirmed etiology	Sepsis, systemic candidiasis, mycotic keratoconjunctivitis, skin involvement
Case 10, 5 months	No confirmed etiology	Adenovirus infection
Case 12, 16 years	Secondary HLH, EBV	Rotavirus infection
Case 15, 3 years	Secondary HLH, EBV	Neurological impairment
Case 16, 2 months	Primary HLH	Digestive hemorrhage
Case 18, 17 years	Secondary HLH, malaria, and EBV	Multiorgan failure
Case 22, 14 years	Secondary HLH, EBV	Sepsis, hemorrhagic shock, death

We had a survival rate of 69.56%; five patients died, four with progressive disease and one due to complications (see Table 6). Two patients were lost to follow-up. All patients with familial HLH died, and out of the secondary forms, only two were EBV positive. 

**Table 6:** Causes of death HLH: hemophagocytic lymphohistiocytosis; EBV: Epstein-Barr virus

Age/sex	HLH type	Cause of death
13 years, F	Secondary HLH (no cause)	Progressive disease
3 years, M	Secondary HLH (EBV)	Progressive disease
0.2 years, M	Familial HLH (UNC13D mutation)	Progressive disease
10 years, F	Familial HLH (STXBP2, EBV)	Progressive disease
14 years, F	Secondary HLH (HVB)	Sepsis, hemorrhagic shock

## Discussion

HLH is a challenging disease to the physician due to its complex mechanism of development and very large range of possible etiologies. Signs and symptoms at onset are unspecific, and this is why it leads on several occasions to a late diagnosis, putting patients' lives at risk. Diagnostic criteria have been revised in 2024 by Henter et al. [9] for familial HLH, and two additional strategies to make familial HLH diagnosis were added in patients with signs/symptoms suggestive of HLH: a genetic pathway and a cellular pathway. The clinical pathway is considered to be of utmost importance, and deciding whether to investigate more intensively for familial HLH can rely on this.

Fever and splenomegaly are the two clinical signs that suggest diagnosis, and in our cohort, most of the patients presented with both signs, 86.95% and 73.91%, respectively. Even if not all patients fulfilled the criteria for HLH-2004 regarding cytopenias, all patients had at least one-lineage cytopenia at diagnosis, suggesting that associated clinical symptoms with cytopenias could raise the question of HLH. Anemia and thrombocytopenias are seen in more than 80% of HLH patients [2], as was the case in our population. Patients mostly presented with low hemoglobin levels; only two had normal hemoglobin values according to age. Next in line were the platelet counts, with only five patients presenting a normal platelet count. Regarding WBC, 13 patients had low levels, but interestingly, we also had six with leukocytosis, which is not characteristic, as leukopenia and neutropenia are one of the criteria for HLH [6]. This happened most probably due to associated infections at diagnosis. Although bone marrow hemophagocytosis is not pathognomonic [1] and not essential for the positive diagnosis, all of our patients presented with this criteria.

Hyperferritinemia is seen in most cases of pediatric HLH [5,6], and this was also seen in most of the patients of our studied population, and more so, it was the biological criteria most often present, suggesting its high specificity.

Qiu et al. [14] showed that high levels of triglycerides should alert the physician to search for EBV infection, more so as they are independent risk factors for poor prognosis. Triglycerides were only high in half of the patients tested, and only 5/10 (50%) of EBV-infected patients showed higher levels of triglycerides. In our population, only one of the EBV-infected patients with higher levels of triglycerides had a partial remission, received additional therapy with rituximab, but eventually died of progressive disease.

Other organ involvement has been shown to occur in HLH patients [2,6], and this was proven also in our cohort, where seven patients also presented at diagnosis with organ, hepatic, and renal failure and pancreatic, pulmonary, or skin involvement, proving the multisystemic inflammation that characterizes the disease.

Our cohort was represented by only two confirmed cases of familial HLH, one with UNC13D and one with STXBP2, classified as familial-type HLH 3 and 5 [3,15]. It is important to mention that the exact number of patients with familial HLH might be higher, taking into account the lack of possibilities in testing in all patients during the 18-year period of the study. STXBP2-mutated patients often present with sensorineural hearing loss, bleeding, and severe diarrhea [6], but this was not the case with our patient. Malignancy-associated HLH has been reported in 8% of the cases [15], and in our cohort, it was present in 13% of the cases. Leukemias and lymphomas are the most common malignancies associated with HLH [15-18]; they were also reported in our cohort, but we also had one case with Wilms tumor which is uncommon. Malignancy-associated HLH is known to have a rapidly progressive evolution [15], and balancing both HLH therapy and specific chemotherapy is challenging for the physician [16]. Our patients all survived the malignancy-associated HLH.

It is well-known that EBV is one of the most frequent causes for secondary HLH, which was proven also in our cohort [2,6,11,15]. A Japanese study led by Kogawa et al. [19] showed that secondary HLH due to EBV infection has a cure rate of 90%. In our study, 60% (6/10) of EBV-infected patients showed improvement with only induction therapy. Rituximab was used only in patients with confirmed EBV infection, as it is shown in the literature that the addition of rituximab might help these patients improve [1], but in our population, both patients died due to progressive disease. Ruxolitinib has also been shown to have a better efficacy in EBV-infected patients [12], but in our cohort, we did not have the opportunity to use this medication at that timepoint. Zhang et al.'s study [12] showed that monotherapy with ruxolitinib had a lower cure rate, compared to the association of both ruxolitinib and chemotherapy, but even so, there was still a percentage of non-responders suggesting that novel therapies are still necessary.

Malaria is a rare disease in our country, but due to increased travelling, it occurs more and more often. Malaria infection has been described to trigger HLH in several cases [5], but due to the overlapping symptoms of both diseases [5,20], most cases might go undiagnosed. Studies published show that the mortality rate of malaria-associated HLH is lower compared to other infections and less than half of the patients received specific treatment [5]. Our case received only dexamethasone and one administration of etoposide. After the initiation of antimalarial agents, he went into remission without requiring further HLH therapy.

HIV-associated HLH was present in only one case, who responded quickly to induction therapy associated with antiretroviral therapy. Tabaja et al. [7] concluded that chronically active HIV patients are more likely to develop HLH, sustaining the fact that antiretroviral therapy should continue even during active HLH.

High-dose anakinra has proven its efficacy in several severe refractory cases [21,22], but in our cohort, although in the first few days the patient showed improvement, she eventually had a severe relapse with multiorgan failure and died.

Mahlaoui et al. [23] showed in their study that the association of ATG to initiation therapy increased the response rate but recurrence was still high. In our population, we only used ATG in two cases; both were with familial HLH and did not achieve remission after treatment.

Although HSCT is a clear indication for primary HLH, none of the familial HLH cases managed to benefit from HSCT. The patient with STXBP2 heterozygosity and EBV infection did not manage to go into remission. The patient with UNC13D mutation struggled with the disease during the COVID-19 pandemic, when access to medical care was a lot more difficult and the donors who were found refused to come, leading to a severe relapse after induction therapy with progressive disease and the death of the patient.

HLH is known to have a high mortality rate [11] of approximately 50%, but our cohort, even with limited access to some tests or even targeted therapy, achieved a survival rate of 69.56%. Four out of five deaths were caused by progressive disease; only one patient died due to associated infectious complications. Out of the four patients who died due to progressive disease, only one was not tested for genetic alterations, which might have led to a possible insufficient therapeutic approach. More so, it is important to mention that the lack of genetic testing in all patients might have altered the classification of HLH types in our population.

Our study comprises an 18-year experience period of the disease, with variable diagnostic and treatment options throughout the years. Nonetheless, it is important to outline that the small number of patients and the lack of genetic testing for every patient with no confirmed cause might cause a bias in interpretation. More so, the small cohort also limits the generalizability of the conclusions regarding treatment efficacy.

## Conclusions

HLH is a very challenging disease for any physician. Numerous causes have been reported, starting with genetic predisposition and ending with a long list of secondary causes, making the choice of treatment for this disease quite difficult.

Protocols are available for first-line therapies, namely, the HLH-2004 protocol, which was used in all our patients. The majority of patients had a good evolution with induction therapy. There were still patients with relapsed and refractory forms, and in these situations, the treatment is up to the physician's choice according to each individual. Several targeted therapeutic choices are now available, such as emapalumab for primary refractory HLH and anakinra or ruxolitinib for secondary refractory forms. In our cohort, they did not prove to be efficacious, but due to the small number of patients, we cannot draw any specific conclusions regarding treatment outcome with these agents. 

Our study is a descriptive study that analyzes HLH over a long period of time in a single center, with times of limited resources and times when genetic testing was available. It offers a good insight into a personal experience, but it has important limitations regarding potential conclusions towards classification and therapeutic outcome due to the small population.

Clinical trials on bigger cohorts and studies with novel therapies need to be led in order to give patients with refractory disease a chance at survival.
